# Safety and immunogenicity of varied doses of R21/Matrix-M™ vaccine at three years follow-up: A phase 1b age de-escalation, dose-escalation trial in adults, children, and infants in Kilifi-Kenya

**DOI:** 10.12688/wellcomeopenres.19795.1

**Published:** 2023-10-12

**Authors:** Samuel Sang, Mehreen S. Datoo, Edward Otieno, Charles Muiruri, Duncan Bellamy, Emmaloise Gathuri, Omar Ngoto, Janet Musembi, Sam Provstgaard-Morys, Lisa Stockdale, Jeremy Aboagye, Daniel Woods, Alison Lawrie, Racheal Roberts, Kelvias Keter, Domtila Kimani, Francis Ndungu, Melissa Kapulu, Irene Njau, Benedict Orindi, Katie J. Ewer, Adrian V.S. Hill, Philip Bejon, Mainga Hamaluba

**Affiliations:** 1 KEMRI-Wellcome Trust Research Programme, Kilifi, Kilifi, 80108, Kenya; 2 The Jenner Instituite, Centre for Vaccinology and Tropical Medicine, University of Oxford and NIHR Biomedical Researcg Centre, Oxford, OX3 7LA, UK; 3 University of Oxford, The Jenner Instituite Laboratories, Old Campus Research Building (ORCB), Roosevelt Drive, OX3 7DQ, UK; 4 Centre for Tropical Medicine and Global Health, South Parks Road, Oxford, OX1 3SY, UK

**Keywords:** R21, Matrix-M™, Adults, children, infants, Kenya, falciparum, age de-escalation, dose escalation, safety, immunogenicity, CSP

## Abstract

**Background:**

Falciparum malaria remains a global health problem. Two vaccines, based on the circumsporozoite antigen, are available. RTS, S/AS01 was recommended for use in 2021 following the advice of the World Health Organisation (WHO) Strategic Advisory Group of Experts (SAGE) on Immunization and WHO Malaria Policy Advisory Group (MPAG). It has since been pre-qualified in 2022 by the WHO. R21 is similar to RTS, S/AS01, and recently licensed in Nigeria, Ghana and Burkina Faso following Phase 3 trial results.

**Methods:**

We conducted a Phase 1b age de-escalation, dose escalation bridging study after a change in the manufacturing process for R21. We recruited healthy adults and children and used a three dose primary vaccination series with a booster dose at 1–2 years. Variable doses of R21 and adjuvant (Matrix-M ™) were administered at 10µgR21/50 µg Matrix-M™, 5µgR21/25µg Matrix-M™ and 5µgR21/50µg Matrix-M™ to 20 adults, 20 children, and 51 infants.

**Results:**

Self-limiting adverse events were reported relating to the injection site and mild systemic symptoms. Two serious adverse events were reported, neither linked to vaccination. High levels of IgG antibodies to the circumsporozoite antigen were induced, and geometric mean titres in infants, the target group, were 1.1 (0.9 to 1.3) EU/mL at day 0, 10175 (7724 to 13404) EU/mL at day 84 and (following a booster dose at day 421) 6792 (5310 to 8687) EU/mL at day 456.

**Conclusion:**

R21/Matrix-M™ is safe, and immunogenic when given at varied doses with the peak immune response seen in infants 28 days after a three dose primary vaccination series given four weeks apart. Antibody responses were restored 28 days after a 4 ^th^ dose given one year post a three dose primary series in the young children and infants.

**Registration:**

Clinicaltrials.gov (NCT03580824; 9 ^th^ of July 2018; Pan African Clinical Trials Registry (PACTR202105682956280; 17 ^th^ May 2021).

## Introduction

Malaria remains a major public health burden with 247 million malaria cases estimated in 2021, and 95% of these occurring in the African region ^1^. Malaria can manifest as mild or more severe disease, with severe cases leading to an estimated 619,000 deaths per annum globally ^1^. Vulnerable groups outside of early childhood include women with approximately one-third of the 40 million pregnancies in Africa affected. Malaria in pregnancy and early childhood is associated with anemia ^2– 4^, severe disease, poor birth outcomes (low birth weight, prematurity, and stillbirth), and has an adverse socio-economic impact. Until recently, public health measures targeting malaria control and elimination were primarily limited to long-lasting insecticide-treated bed nets, intermittent preventive treatment, and chemoprevention. A vaccine targeting one of the key life stages of the *P. falciparum* parasite is needed that dramatically reduces the incidence of blood stage infection clinical malaria (e.g., 90% over 12 months of follow up post immunization) ^5^. Pre-erythrocytic vaccines target antigens from the *P. falciparum* sporozoite. They induce antibodies against surface antigens that clear sporozoites from the skin or bloodstream or block the invasion of hepatocytes. Of the 24 vaccine candidates in active clinical development, 38% target the pre-erythrocytic stage (CSP) with R21 being the most advanced in clinical development ^6^.

Recommendation by the World Health Organisation (WHO) for the RTS, S/AS01 vaccine to be used in October 2021 and pre-qualification were both major milestones in advancing malaria control. However, additional measures including highly effective vaccines will be required to meet malaria control targets to reduce global malaria incidence and mortality by at least 90% by 2030 ^7^. R21 is a circumsporozoite protein (CSP) vaccine ^8^, similar to RTS,S/AS01 and adjuvanted with Matrix M™, a naturally occurring saponin from *Quillaja Saponaria*. R21 was designed by the Jenner Institute, University of Oxford using the C-Tag affinity purification process (R21c) ^9^ at The Clinical Biomanufacturing Facility (CBF), UK. Addition of the C-tag was necessitated to overcome technical difficulties in the purification process. Evaluation of R21c in phase 1 trials in the United Kingdom and Burkina Faso demonstrated the R21c vaccine to be safe and immunogenic with less reactogenicity at comparable doses in West African adults ^10^. Due to a change in the manufacturing process on transfer to Serum Institute of India, bridging phase 1 results in adults and children were required. The changes included removal of the additional four amino acids E-P-E-A, comprising the C-tag sequence and expression of the R21 protein in *Hansenula polymorphaat* rather than *Pichia pastoris*. Safety and immunogenicity data following the primary series of this phase 1b trial informed the progression and dose choice to ongoing phase 2 ^11, 12^ and 3 trials. Results reported here are following completion of the phase 1b, where a booster (4 ^th^ dose) was given up to two years following a three dose primary series and follow-up was for 12 months post boosting.

## Methods

### Study design and participants

We conducted a phase 1b, open-label, age de-escalation, dose escalation, trial to evaluate the safety and immunogenicity of R21/Matrix-M™ in healthy Kenyan adults; aged 18–45 years (group 1) then children aged 1–5 years (group 2) and finally infants 5 months to ˂1 year (group 3). Participants were recruited from Junju village within the Kilifi Health and Demographic Surveillance System (KHDSS) ^13^.

The trial was conducted in accordance with the International Conference on Harmonisation (ICH)-Good Clinical Practice (GCP) guidelines ^14^. Ethical approval was obtained from the Kenya Medical Research Institute (KEMRI) Scientific and Ethics Review Unit (CGMR-C/116/3711) on the 26 ^th^ of September 2018 and the Oxford Tropical Research Ethics Committee (OxTREC-6-18) on 27 ^th^ September 2018, and regulatory approval from the Pharmacy and Poisons Board of Kenya (PPB/ECCT/18/12/01/2019) on 18 ^th^ March 2019. Participants were given ample time to consider participation. Informed consent was documented by signature or thumbprint by all participating adults and parents or authorised guardians of children and infants before screening for eligibility. Enrolment was after a test of understanding where a score of >90% was required prior to fully informed consent. 

Stakeholder engagement was conducted, and all approvals were in place prior to recruitment. Recruitment occurred following community meetings and in participants’ homes. All participants were assessed with a screening questionnaire, clinical assessment, safety bloods, and a viral (Human Immunodeficiency Virus- HIV, Hepatitis B, and C) and haemoglobinopathy screen to determine enrolment eligibility. Urine samples were taken in adults to assess for diabetes in this cohort and pregnancy in women. Full trial screening and eligibility criteria are included in the trial protocol ^15^.

R21 is a pre-erythrocytic protein-in-adjuvant malaria vaccine candidate. It is adjuvanted with Matrix-M™ based on the circumsporozoite protein (CSP) produced by using recombinant Hepatitis B surface antigen (HBsAg) particles expressing the central NANP repeat and the C-terminus. It spontaneously forms a particle, but without the need for excess Hepatitis B antigen required for particle formation with RTS, S/AS01.

### Study procedures

Potential participants were screened until the target enrolment was reached based on eligibility criteria including age. Participants were sequentially allocated to one of nine age de-escalating and dose-escalating groups. All participants received a three-dose primary series of vaccinations four weeks apart followed by a 4 ^th^ dose. The 4 ^th^ dose was administered two years after completion of the primary vaccinations in adults and children and one year after primary series in infants. All four vaccinations were at the same dose. Follow-up was 12 months after the 4 ^th^ dose in all groups. R21 was thawed to room temperature then administered intramuscularly after mixing with Matrix-M™. One of three doses was administered: 10µgR21/50µg Matrix-M™ (Groups 1A, 1B, 2B, 3B or 3D), 5µgR21/25µg Matrix-M™ (2A, 3A and 3C) or 5µgR21/50µg Matrix-M™ (3E). Vaccination was into the non-dominant deltoid for adults and children and the right arm for infants. Participants were observed for one hour post-vaccination and adverse events solicited in the week following vaccination using diary cards. Diary card completion was by field workers in participants’ homes for the primary series and over the phone following 4 ^th^ dose due to COVID-19 pandemic restrictions. During the pandemic, following an amendment, participants were invited to attend the study clinic in the event of an adverse event (AE) of severity grade 3 or higher, or where clarification was required of AE grading. Unsolicited adverse events were collected in the 28 days after vaccination. Post-enrolment follow-up visits were conducted on days 28, 42, 56, 84, 238, 421, 456, 786, 814 and 1151. Additionally, safety bloods were taken on days two and seven post-primary series vaccinations. Safety oversight was by two local safety monitors and an independent Data Safety Monitoring Board (DSMB). Decisions to progress within and across groups were determined by the DSMB. Following an initial introductory meeting, the DSMB had six scheduled meetings. Scheduled reviews included cumulative adverse events for all vaccinees. Each group had a sentinel cohort with infants receiving variable doses and the adult doses fixed. DSMB meeting 1 occurred 72 hours after the first participant in group 1A was vaccinated. DSMB meeting 2 occurred no earlier than three weeks after the first dose was administered to the first participants in group 1B, DSMB meeting 3 occurred 10 days after the first dosing of group 2A, DSMB meeting 4 at three weeks after the dosing of the first participant in group 2B. DSMB meeting 5 was 10 days after the first vaccination in group 3A and DSMB meeting 6 was 10 days after the first dose in group 3B. Groups 3C, 3D, and 3E were vaccinated concurrently 10 days following the first vaccination in group 3B and review of cumulative safety data. One unscheduled DSMB meeting was held to review a rising alanine transaminase (ALT) in an adult. Stopping rules included one or more participants experiencing a SAE related to the investigational products or the occurrence of a suspected unexpected serious adverse reaction (SUSAR).

### IgG enzyme-linked immunosorbent assay (ELISA)

Anti-NANP antibodies were measured by ELISA just prior to and 28 days post each vaccination (Baseline, days 28, 56, 84, 421,456,786, 814). IgG responses were also ascertained seven days post first vaccination (Day 7). Additional immunology bleeds occurred 12 and 24 months post primary series (days 421 and 786) and one year post 4 ^th^ dose (days 786 and 1151). Additional immunogenicity timepoints were assessed at 42 days after first vaccination and six months post primary series (Day 238). ELISA methods used were as previously described ^10, 16^. Briefly, 96-well plates (NUNC) were coated with a synthetic peptide comprising 6 repeats of the amino acid sequence NANP (ProImmune) at a concentration of 0.2 μg/mL. After incubation and washing with PBS with 0.05% Tween, wells were blocked with casein (Sigma) for at room temperature for 1 hour. After washing, samples (diluted at between 1:100 and 1:5000) were added in duplicate and incubated at room temperature for 2 hours. After washing, alkaline phosphatase conjugated goat anti-human IgG (Sigma) secondary antibody was added at a dilution of 1:1000 and incubated at room temperature for 1 hour. After washing, P nitrophenyl phosphate (Sigma) and diethanolamine substrate (Pierce) buffer was added and the plate read at 405nm using Gen 5 software. A reference pool of positive serum formed a standard curve on each plate and was used to calculate ELISA Units (EU) for each. An internal control was included on each plate to standardise between assays.

### Objectives and outcomes

The primary objective was to evaluate the safety and tolerability of R21 with the adjuvant Matrix™ in healthy adults then children and finally infants. The secondary objectives were to assess the cellular and humoral immunogenicity of R21 with the adjuvant Matrix-M™ in healthy adults, children, and infants. Outcomes included the occurrence of solicited local and systemic reactogenicity in the seven days following vaccination, unsolicited adverse events in the 28 days after vaccination, changes in baseline laboratory measures and the occurrence of SAEs throughout the study. Secondary outcomes included the comparison of antibody responses and longevity of responses across dose groups up to 25 months following booster vaccinations.

### Statistical analysis

Descriptive statistics were used to summarise all parameters. The analyses were based on an intention-to-treat population, which included all participants who received at least one vaccination dose. Safety data were summarised as the number of participants that experienced any event after vaccination in the study group and as a percentage of the total number of participants within the group. Unsolicited AEs were categorised according to the Medical Dictionary for Regulatory Activities preferred terms (PT). We estimated the geometric means of the IgG antibody titres (GMTs) against the NANP at multiple time points, together with their 95% CIs separately for each study group. Log-transformed anti-NANP IgG titres at day 84 were compared across age, dose and age-dose groups using one-way analysis of variance (ANOVA). Significant ANOVA results were followed by a Tukey post-hoc test. To assess the impact of boosting we estimated GMT fold change (GMFC) at days 421 and 456 relative to day 84 and performed a paired *t*-test within each group. Efforts were made during the study to minimise missing data occurrence. At the analysis stage, numbers (percentages) of incomplete data were reported. Missing data were not imputed in any way. Analyses were performed using Stata version 17.0. Stata Statistical Software: Release 17. College Station, TX: StataCorp LLC, RRID:SCR_012763 was used ( http://www.stata.com). R software version 3.6.2 (R Core Team, 2019, https://www.r-project.org) was also used. Figures were produced using GraphPad Prism version 9.4.0 (GraphPad Software, San Diego, California USA, www.graphpad.com).

## Results

The study was conducted between 28 ^th^ April 2019 and 14 ^th^ June 2022. Primary series and booster vaccinations were complete by December 2019 and June 2021, respectively. The trial protocol was amended to allow for a delayed fourth dose in groups 1 and 2 due to the COVID-19 pandemic.

**Figure 1.**
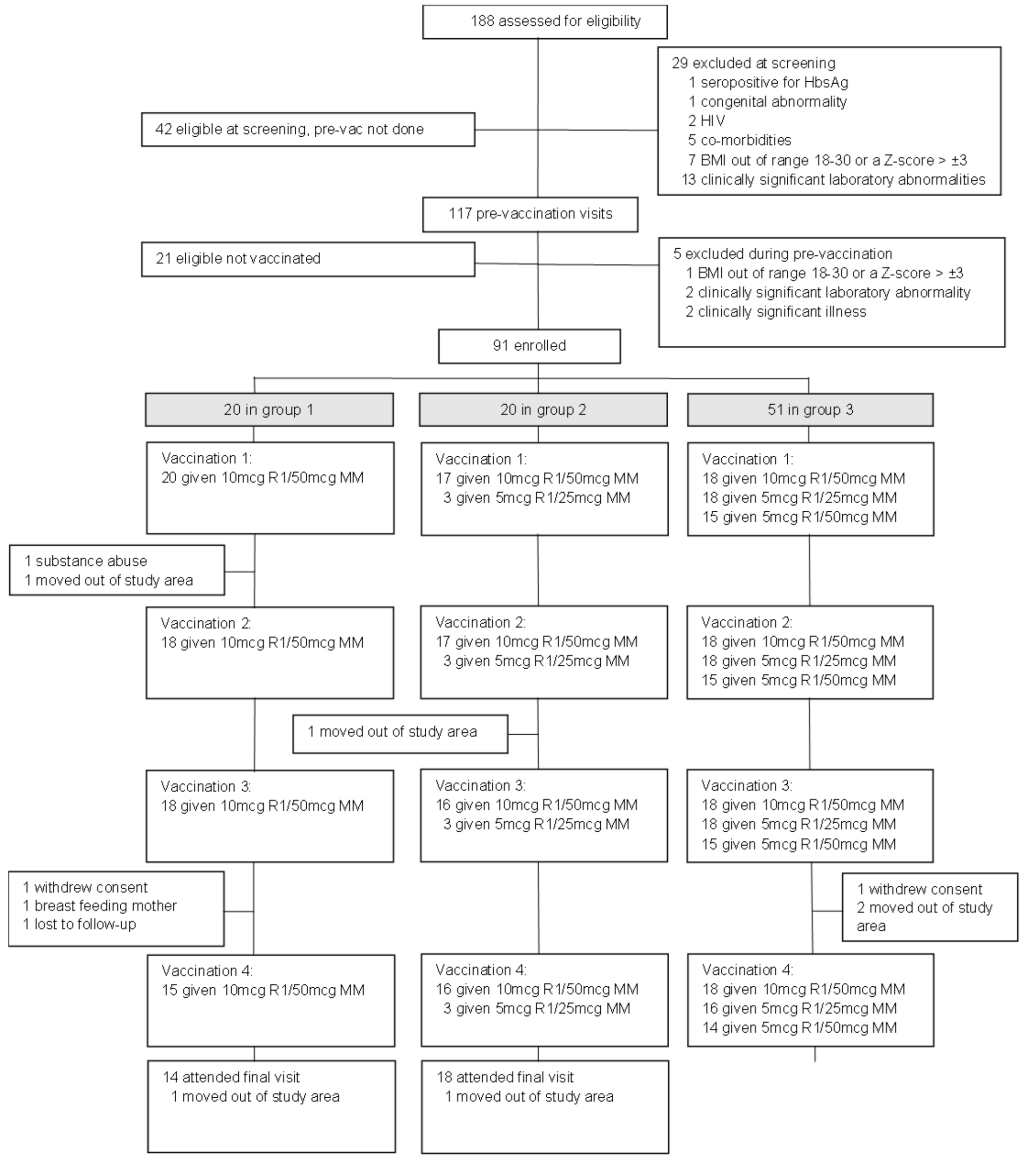
Study profile [CONSORT diagram].

One hundred and eighty-eight participants were screened, 97 were excluded, and 91 were enrolled and received at least one vaccination ( Figure 1). Twenty adults were enrolled in group 1, 20 children to group 2, and 51 infants to group 3. Eighty-eight participants completed primary series vaccinations, with 18 participants in group 1, 19 in group 2, and 51 in group 3. In group 1, two participants were withdrawn before their second vaccination. One participant moved out of the study area and an undisclosed history of substance abuse was uncovered in the second participant. One was excluded after the fourth vaccination as they moved out of the study area. In group 2, one child was withdrawn following second vaccination as the child was no longer resident in the study area. One child was excluded after booster vaccination due to moving out of the study area. In group 3, three infants were withdrawn before their fourth vaccination: two had moved out of the study area and one withdrew consent. After the fourth vaccination, two infants withdrew consent not due to adverse event, two infants moved out of the study area, and one infant was lost to follow-up. All enrolled participants were included in the safety and immunogenicity analyses. Three in four of those in group 1 were males. Groups 2 and 3 were generally balanced with respect to gender. The median age at enrolment was 27 years (IQR 25–31) for group 1, 35 months (IQR 27–49) for group 2 and eight months (IQR 6–9) for group 3 ( Table 1). Five (25%) participants in group 1, one (5%) in group 2, and nine (18%) in group 3 had sickle cell trait. The median interval between primary and booster vaccination was 23 months for group 1, 22 months for group 2 and 12 months for group 3.

**Table 1.** Demographic and baseline characteristics.

	Group 1	Group 2	Group 3
Number enrolled	20	20	51
Gender			
Male	15 (75%)	9 (45%)	24 (47%)
Female	5 (5%)	11 (55%)	27 (53%)
Age in years, median (IQR)	27.0 (24.8–31.3)	–	–
Age in months, median (IQR)	–	34.5 (27.0-49.0)	8.0 (6.0–9.0)
BMI, kg/m ^2^, median (IQR)	22.1 20.5–22.9)	–	–
Z score	-	1.0	-1.0

### Safety assessments

***Solicited local adverse events.*** A total of 1077 adverse events were reported, with each participant reporting at least an AE. One hundred and forty-five AEs were reported for group 1, 199 for group 2 and 733 for group 3 ( Table 2). 

**Table 2.** Summary of all adverse events for consented subjects.

	Group 1	Group 2	Group 3	Overall N=91
**Number of AEs reported**	145	199	733	1077
**Number of Subjects with AEs [1]**	20	20	51	91
**Number of SAE's reported**	0	0	2	2
**Number of subjects with SAEs [1]**	0	0	2	2
Number of AEs by severity *				
Mild	137 (94.5%)	86 (43.2%)	682 (93%)	1005 (93.3%)
Moderate	8 (5.5%)	12 (6%)	44 (6%)	64 (5.9%)
Severe	0 (0%)	1 (<1%)	7 (1%)	8 (1%)
Potentially life-threatening	0 (0%)	0 (0%)	0 (0%)	0 (0%)
Subjects with AEs by Severity [2] **				
Mild	20 (100%)	20 (100%)	51 (100%)	91 (100%)
Moderate	7 (35%)	7 (35%)	26 (51%)	40 (44%)
Severe	0 (0%)	1 (5%)	6 (12%)	7 (8%)
Potentially life-threatening	0 (0%)	0 (0%)	0 (0%)	0 (0%)

[1] Subjects who experience one or more AEs or SAEs are counted only once [2] Subjects are counted only once within a particular severity grade *Percentages are based on number of AEs reported in each study group **Percentages are based on N for each treatment arm

Injection site pain occurred in 12 (60%) of 20 participants in group 1 and two (10%) of 20 participants in group 2 after the first vaccination ( Table 3). Solicited injection site pain occurred in 13 (72%) of 18 participants in group 1, five (25%) of 20 participants in group 2, and three (6%) of 51 participants in group 3 after the second vaccination. Pain occurred in two (11%) of 18 participants in group 1 and three (16%) of 19 participants in group 2 after the third vaccination. No pain was reported in group 1 after the fourth vaccination dose. Participants reporting solicited pain at the injection site decreased as vaccinations progressed in groups 2 and 3. Other local AEs occurring in small proportions of adults (group 1) were induration, injection site itch and injection site warmth ( Table 3; Table S1 ^15^). All solicited local AEs were graded as either mild or moderate (Table S1 ^15^), with 64 (92.8%) of 69 AEs in group 1, 13 (92.8%) of 14 AEs in group 2, and 11 (91.7%) of 12 AEs in group 3 self-resolving. Seven hundred and thirty-three AEs were documented in all group 3 infants, 256 in those that received 10µgR21/50µg Matrix-M™ (groups 3B/D), 250 in 3A/3C (5µgR21/25µg Matrix-M™) and 227 in group 3E (5µgR21/50µg Matrix-M™) (Table S4 ^15^).

***Solicited systemic adverse events.*** One week following vaccination, 11 solicited systemic adverse event experiences were reported in group 1, 28 in group 2 and 78 in group 3. Chills occurred in two (10%) of the 20 participants in group 1 after the first vaccination. Myalgia, arthralgia, and fatigue occurred in one (5%) of the 20 participants in group 1 after the first vaccination. Other than headache and fatigue, no solicited systemic AEs were observed in group 1 after the second, third and fourth vaccinations. No participant in group 1 reported fever ( Table 3). Most systemic solicited AEs (73%, n=8) in group 1 were classified as related to the investigational product. Fever was the most frequent solicited systemic adverse event in groups 2 and 3, accounting for 20 (71%) of 28 solicited AEs in group 2 and 59 (76%) of 78 AEs in group 3, with the proportion of participants reporting fever increasing with the vaccination number. One participant in group 2 reported severe fever after the third vaccination and one participant in group 3 reported severe fever after the second, third and fourth vaccinations; all other adverse events were graded as either mild or moderate ( Table 3; Table S1 ^15^). Eight (73%) solicited systemic AEs in group 1 and all AEs in groups 2 and 3 were self-resolving.

**Table 3.** Adverse events by group and number of vaccination doses *.

	Group 1	Group 2	Group 3
**Local adverse** ** event**			
Induration			
1	2 (10%)	_	_
2	1 (6%)	_	_
3	0 (0%)	_	_
4	0 (0%)	_	_
Injection site pain			
1	12 (60%)	2 (10%)	0 (0%)
2	13 (72%)	5 (25%)	3 (6%)
3	2 (11%)	3 (16%)	0 (0%)
4	0 (0%)	1 (5%)	0 (0%)
Injection site itch			
1	2 (10%)	0 (0%)	0 (0%)
2	2 (11%)	0 (0%)	0 (0%)
3	1 (6%)	0 (0%)	0 (0%)
4	0 (0%)	0 (0%)	0 (0%)
Injection site warmth			
1	3 (15%)	0 (0%)	0 (0%)
2	4 (22%)	0 (0%)	1 (2%)
3	2 (11%)	0 (0%)	0 (0%)
4	0 (0%)	0 (0%)	0 (0%)
Swelling			
1	_	0 (0%)	0 (0%)
2	_	0 (0%)	2 (4%)
3	_	1 (5%)	1 (2%)
4	_	2 (11%)	3 (6%)
**Systemic adverse event**			
Fever			
1	0 (0%)	0 (0%)	3 (6%)
2	0 (0%)	7 (35%)	16 (31%)
3	0 (0%)	9 (47%)	18 (35%)
4	0 (0%)	2 (11%)	18 (38%)
Chills			
1	2 (10%)	_	_
2	0 (0%)	_	_
3	0 (0%)	_	_
4	0 (0%)	_	_
Myalgia			
1	1 (5%)	_	_
2	0 (0%)	_	_
3	0 (0%)	_	_
4	0 (0%)	_	_
Arthralgia			
1	1 (5%)	_	_
2	0 (0%)	_	_
3	0 (0%)	_	_
4	0 (0%)	_	_
Headache			
1	0 (0%)	_	_
2	1 (6%)	_	_
3	1 (6%)	_	_
4	1 (7%)	_	_
Fatigue			
1	1 (5%)	_	_
2	1 (6%)	_	_
3	0 (0%)	_	_
4	0 (0%)	_	_
Loss of appetite			
1	_	0 (0%)	2 (4%)
2	_	1 (5%)	4 (8%)
3	_	2 (11%)	2 (4%)
4	_	1 (5%)	3 (6%)
Irritability			
1	_	0 (0%)	2 (4%)
2	_	0 (0%)	3 (6%)
3	_	2 (11%)	0 (0%)
4	_	0 (0%)	1 (2%)
Drowsiness			
1	_	0 (0%)	0 (0%)
2	_	0 (0%)	1 (2%)
3	_	1 (5%)	0 (0%)
4	_	0 (0%)	1 (2%)

*Data are number (percentage) participants in each group at vaccination 1, 2, 3 and 4. A dash (–) means that a particular event was not assessed for that group. All solicited local and systemic adverse events were collected for 7 days after each vaccination. In group 1, 20 participants received the first vaccination dose, 18 participants received a second and a third dose, and 15 participants received a fourth dose. In group 2, 20 participants received the first and a second vaccination dose, and 19 participants received a third and a fourth dose. 51 participants in group 3 received the first, a second and a third vaccination dose, and 48 participants received a fourth dose.

***Laboratory safety bloods.*** Transient changes in safety bloods were seen during the study. All clinically significant results had settled at the point of the final study visit except in one participant who was withdrawn due to substance misuse (alcohol) associated with high ALTs.

***Unsolicited adverse events within 28 days of vaccination.*** There was a total of 396 unsolicited AEs (36 in group 1, 74 in group 2, and 286 in group 3) in the month following each vaccination. Abnormal safety laboratory results were found in 22.5% (89/396). Three hundred and sixty-eight (92.9%) of the unsolicited AEs were mild in nature. A total of 94.2% (373/396) were classified as unrelated to the investigational product and almost all (389/396) resolved. Twenty-seven cases of malaria, none of which were severe, were reported within 28 days after vaccination, while 104 malaria cases were reported throughout the study across groups.

***Serious adverse events.*** There were two serious adverse events during the study requiring hospitalisation, neither related to vaccination. The first SAE was in a 13-month-old who was diagnosed with a lower respiratory tract infection. The second SAE was also a lower respiratory tract infection following seven days of symptoms (cough, intermittent fevers associated with one febrile convulsion, and shortness of breathe). Both resolved uneventfully. There was one pregnancy reported (conception approximately 3.5 months post third vaccination) that resulted in a healthy female infant as assessed at birth, four and seven months of age.

### NANP-specific IgG responses

**Figure 2.**
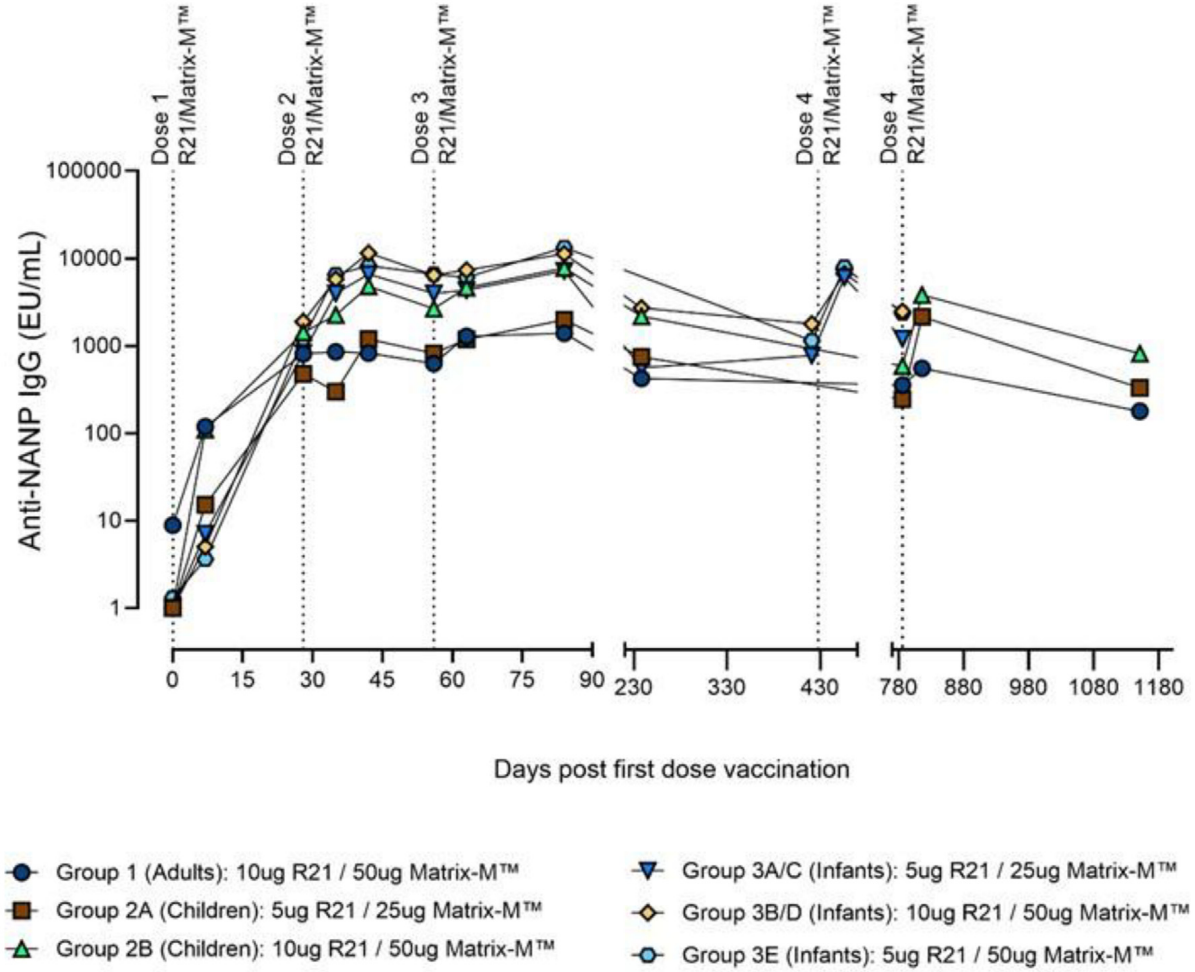
Geometric mean for NANP IgG antibody titres by age at dose administered.

Peak NANP-specific IgG responses were seen at day 84 (28 days following the primary series of vaccinations) across dose and age groups ( Figure 2). The highest GMTs seen were in group 3E infants who received 5μgR21/50μg Matrix-M™ recording a GMT of 13,279 (95% CI 7366–23,942) at day 84, almost ten times higher than adults who received 10µgR21/50µg Matrix-M™ with the lowest GMT of 1392 (95% CI 984–1969) (p<0.001; Table 4). Using one-way ANOVA NANP IgG titres at day 84 varied significantly by age (F=28.1, df=2, 84; p<0.001), dose (F=4.1, df=2, 82; p=0.019), and age-dose (F=13.9, df=5, 81; p<0.001). However, when the analysis by dose was restricted to the infant groups, these differences by dose were not statistically significant by the Tukey post-hoc test (Table S2 ^15^). All groups elicited high GMTs 28 days post primary series which declined significantly prior to boosting one and two years later (days 421 and 786) except in group 2A (Table S3 ^15^). At day 421 GMFC from day 84 were 0.25 (95% CI 0.15–0.44; p<0.001) in group 1 and 0.12 (95% CI 0.08–0.18; p<0.01) in group 3 (Table S3 ^15^). At day 456 although the GMFC was 0.7 (95% CI 0.5 to 0.9) in group 3, for individual dose groups there were no significant decreases in GMTs when compared to day 84 ( Table 4).

**Table 4.** Immunological outcomes by age, dose, and age-dose category.

Group	Number enrolled	Geometric mean titre at day 0	Geometric mean titre at day 84 *	Geometric mean titre at day 456/814 **	GMFC (95% CI) †	Paired †† *t* test pvalue
Age						
Adults (Group 1A/B)	20	7.1 (2.5 to 20.2)	1392 (984 to 1969)	554 (343 to 897)	0.4 (0.3 to 0.7)	0.002
Children (Group 2A/B)	20	1.0 (1.0 to 1.0)	6242 (3579 to 10886)	3493 (2745 to 4445)	0.6 (0.3 to 0.9)	0.028
Infants (Group 3A-E)	51	1.1 (0.9 to 1.3)	10175 (7724 to 13404)	6792 (5310 to 8687)	0.7 (0.5 to 0.9)	0.015
Dose						
10µgR21/50µg MM	53	2.0 (1.3 to 3.2)	4867 (3433 to 6899)	2444 (1690 to 3535)	0.5 (0.4 to 0.7)	<0.001
5µgR21/25µg MM	23	1.0 (1.0 to 1.0)	5948 (3554 to 9955)	5131 (3303 to 7971)	0.8 (0.4 to 1.4)	0.341
5µgR21/50µg MM	15	1.3 (0.7 to 2.3)	13279 (7366 to 23942)	7958 (4662 to 13584)	0.7 (0.4 to 1.3)	0.223
Age-dose						
Adult:10µgR21/50µg MM (1A/B)	20	7.1 (2.5 to 20.2)	1392 (984 to 1969)	554 (343 to 897)	0.4 (0.3 to 0.7)	0.002
Children-5µgR21/25µgMM (2A)	3	1.0 (1.0 to 1.0)	1984 (17 to 238491)	2144 (1047 to 4389)	1.1 (0 to 265.4)	0.957
Children-10µgR21/50µgMM (2B)	17	1.0 (1.0 to 1.0)	7739 (4819 to 12428)	3827 (2960 to 4949)	0.5 (0.3 to 0.7)	0.002
Infants-5µgR21/25µgMM (3A/C)	18	1.0 (1.0 to 1.0)	7219 (4684 to 11126)	6044 (3742 to 9763)	0.7 (0.4 to 1.2)	0.206
Infants-10µgR21/50µgMM (3B/D)	18	1.0 (1.0 to 1.0)	11271 (6995 to 18160)	6661 (4558 to 9735)	0.6 (0.3 to 1.1)	0.111
Infants-5µgR21/50µgMM (3E)	15	1.3 (0.7 to 2.3)	13279 (7366 to 23942)	7958 (4662 to 13584)	0.7 (0.4 to 1.3)	0.223

*One-way ANOVA test for NANP IgG titres at day 84 was significant for age (F=28.1, df=2, 84; p<0.001), dose (F=4.1, df=2, 82; p=0.019), and age-dose (F=13.9, df=5, 81; p<0.001) cohorts. Tukey post-hoc test is presented in Table S2 ^15^. †GMFC is anti-NANP geometric mean titre fold ratio at day 456 relative to day 84. ††P-values comparing anti-NANP IgG titres at days 84 and 456 for each age, dose and age-dose category. ** 28 days post 4 ^th^ dose was day 456 for groups 3, day 814 for groups 1 and 2

After day 28, Anti-NANP IgG reached their lowest levels at days 421 and 786 across groups and doses ( Figure 2). The antibody kinetics in Figure 2 show substantial immune responses to the first vaccination at day 28 for infants and children which peak at day 84 then wane at days 421 and 786. Following a fourth dose of R21/Matrix-M™, however, anti-NANP antibody levels were restored at day 456 and 814 (28 days post 4 ^th^ dose) in groups 2 and 3 [ Figure 2]. The GMTs at day 456 and 814 rose post 4 ^th^ dose to levels similar to those seen at day 84 for all age-dose groups except groups 1A/B and 2A where a decline in GMTs was observed p=0.002 ( Table 4). Immune responses were not assessed in 48 (53%) of 91 participants six months post primary series (day 238). A total of 45/48 of these were due to pandemic restrictions in group 3 infants and three withdrawals.

## Discussion

R21/Matrix-M™ is safe when given as a three dose primary series with a 4 ^th^ dose one to two years later in adults, young children, or infants at a dose of :10μgR21/50μg Matrix-M™, 5µgR21/25µg Matrix-M™ or 5µgR21/50µg Matrix-M™. There were no serious adverse events related to the investigational product and most solicited adverse events were mild to moderate and self-limiting. Systemic adverse events following vaccination were uncommon in Kenyan adults and similar to profiles seen in Burkinabe adults vaccinated with three doses of R21c ^10^. The frequency of adverse events was inversely related to the number of doses administered ( Table 3). Pain was the most frequent AE seen in adults, particularly after the 1 ^st^ and 2 ^nd^ vaccinations. In children, pain and temperature were the most frequent AEs solicited. In vaccinated infants, local adverse events in the seven days following vaccination were infrequent with mild pain being most common in the 1–5 years age group. Mild to moderate temperatures were the most common systemic adverse events irrespective of dose in those aged under five years. AEs were most frequently seen in the infant groups but were mostly mild to moderate and similar in frequency across age-dose groups.

R21 elicited high anti-NANP IgG GMTs following a three dose primary series for all age groups and R21/Matrix-M™ doses received. GMTs were inversely related to age irrespective of dose administered. In the target population, children’s immune responses were optimal in those vaccinated at an earlier age (5 to ˂12 months) with three dose primary series 28 days apart and boosting at 12 months post 3 ^rd^ dose. The optimal dose that induced the maximum response as measured by anti-NANP IgG response was seen in the infants vaccinated with 5μgR21/50μg Matrix-M™. Anti-NANP IgG GMTs in group 3 when 5µgR21/50µg Matrix-M™ was administered were 13,279 (7366–23,942) at day 84 and 6661 (4558–9735) at day 456, the highest in any dose or age group. Infants vaccinated with the higher adjuvant dose had higher GMTs post primary series and booster irrespective of R21 protein concentration. Infants vaccinated with half the protein/adjuvant dose (5µgR21/25µg Matrix-M™ (3A/C) had almost 50% the response seen (GMTs) compared to infants vaccinated in Group 3E (5µgR21/50µg Matrix-M™) at day 84. GMFC at day 456 however in groups 3E and 3A/C were comparable; 0.7 (95% CI 0.4 to 1.3) and 0.7 (95% CI 0.4 to 1.2) respectively ( Table 4). Given that 10µgR21/50µg Matrix-M™ elicited lower GMTs at day 84 than 5µgR21/50µg Matrix-M™ in the infant groups with no appreciable difference in adverse event profile given the group sizes and a GMFC at day 456 of 0.6 (95% CI 0.3 to 1.1) the optimal dose identified was 5µgR21/50µg Matrix-M™ for infants. This dose was not evaluated in group 1 or 2 and group 2A had a small sample size (n=3) making difficult to extrapolate similarities in protein/adjuvant response in these age groups. Further studies are required to examine the immune responses of different R21 protein/Matrix-M™ concentrations in older children. The 5µgR21/50µg Matrix-M™ dose is now under evaluation in a phase 3 trial with five sites in Kenya, Tanzania, Burkina Faso, and Mali.

We observed a decay in anti-NANP IgG titres pre- boosting (days 421 and 786) most notable in the adults and children who were sampled two years post their 3 ^rd^ vaccination. There was, however, evidence of a boosting response when pre and post boosting GMTs were compared with GMTs rising seven-fold in groups 1 and 2. Although the boosting response in adults was modest, boosting was more immunogenic in children and in infants. A three dose primary series regime with one or more boosters may be an effective approach for sustained protection in early childhood. Further studies assessing antibody function and vaccine efficacy will help determine whether the apparent lower boosting in adults indicates reduced responsiveness due to multiple previous exposure to malaria. There were no solicited adverse events following the 4 ^th^ vaccination in adults which may be in keeping with a sub-optimal immune boosting response observed at day 84. Baseline antibodies were detectable in group 1 adults. Anti-NANP IgG GMTs at day seven were lower in the infants than the children and adults, this could be due to an anamnestic response in the 1 to 5 year olds and adults compared to the infants.

Retention of study participants was good despite the COVID-19 pandemic with only one participant lost to follow-up although eight were withdrawn as they no longer fulfilled the eligibility criteria. Limitations included the small size of the study and the open label design. Due to the COVID-19 pandemic restrictions the methods used for collection of data were also modified at the point of boosting to minimise risks of SARS-CoV-2 transmission to participants and staff and to comply with public health directives. Moderate and severe AEs are unlikely to have been missed due to daily telephone contact in the week post immunization and clinic attendance for grade 3 or 4 AEs. All SAEs were captured in hospital as recruitment was in the KHDSS where clinical episodes are linked to community members.

## Conclusions

These data demonstrate that R21/Matrix-M™ is safe and should be evaluated in larger randomised controlled clinical trials to ascertain further safety, reactogenicity, immunogenicity and to assess its efficacy in a broader African paediatric and adult population.

## Consent

Written informed consent for publication of the participants’ details was obtained from the participants and the parents or guardian of the participant.

## Data Availability

Harvard Dataverse: Underlying data for ‘A Phase 1b, open-label, age de-escalation, dose-escalation study to evaluate the safety and immunogenicity of different doses of a candidate malaria vaccine; adjuvanted R21(R21/MM) in adults, young children and infants in Kilifi, Kenya’, https://doi.org/10.7910/DVN/IRGZ35 ^15^ Due to the small numbers of participants and difficulty in maintaining participant privacy, the data underlying this research is restricted. De-identified, individual participant data that underlie this article, along with a data dictionary describing variables in the dataset, will be made available to researchers whose proposed purpose of use is approved by the Kenya Medical Research Institute Wellcome Trust Research Programme (KWTRP) data access committee. Essential documents such as the protocol, statistical analysis plan an informed consent forms and safety data can be accessed using the KWTRP Harvard Dataverse repository ^15^. All other data is managed on request by the KWTRP data management committee in order to maintain participant privacy due to the small sample size. Requests can be made for data by emailing dgc@kemri-wellcome.org. There will be no restrictions on analysis; all requests will be evaluated by the data access committee for a decision in line with consent for data sharing and findability, accessibility, interoperability and reusability (known as FAIR) principles. Harvard Dataverse: Extended data for ‘A Phase 1b, open-label, age de-escalation, dose-escalation study to evaluate the safety and immunogenicity of different doses of a candidate malaria vaccine; adjuvanted R21(R21/MM) in adults, young children and infants in Kilifi, Kenya’, https://doi.org/10.7910/DVN/IRGZ35 ^15^ This project contains the following extended data: 210125_VAC073 Protocol Version 2.3.pdf 210204_Adendum 1.2_English Adult ICF_ Version 1.8.pdf Child and Infant ENGLISH ICF version 1.8_Highlighted 27March.pdf VAC073 _Data_Codebook.pdf VAC073(R21-MM)_Statistical_Analytical Plan (003).pdf VAC073_Data_Readme.txt Solicitedaes.tab_csv Supplementary tables Harvard Dataverse: CONSORT checklist for ‘Safety and immunogenicity of varied doses of R21/Matrix-M™ vaccine at three years follow-up: A phase 1b age de-escalation, dose-escalation trial in adults, children, and infants in Kilifi-Kenya’, https://doi.org/10.7910/DVN/IRGZ35 ^15^ Data are available under the terms of the Creative Commons Attribution 4.0 International license (CC-BY 4.0)
