# Supplementary material for: Safety and immunogenicity of varied doses of R21/Matrix-M™ vaccine at three years follow-up: A phase 1b age de-escalation, dose-escalation trial in adults, children, and infants in Kilifi-Kenya
Source: Wellcome Open Res. 2023 Oct 12;8:450. [Version 1] doi: 10.12688/wellcomeopenres.19795.1 (PMC11134133; doi:10.12688/wellcomeopenres.19795.1)
Supplement: Peer review history

# Reviewer report: Reviewer response for version 1

**DOI:** 10.21956/wellcomeopenres.21926.r81717

**Published:** 2024-05-28

**Authors:** Irene S Soares

**Affiliations:** 1 Department of Clinical and Toxicological Analyses, School of Pharmaceutical Sciences, University of São Paulo, São Paulo, Brazil

The most advanced *P. falciparum* circumsporozoite protein (PfCSP)-based malaria vaccine, RTS,S/AS01 (RTS,S), confers partial protection but with antibody titers that wane relatively rapidly, highlighting the need to elicit more potent and durable antibody responses. This malaria vaccine was recommended by the WHO for use in African countries in October 2021. R21/Matrix-M™ is similar to RTS,S/AS01 and was recently licensed in Nigeria, Ghana, and Burkina Faso following Phase 3 trial results.

 The manuscript by Sang et al. reports on a phase 1b age de-escalation, dose-escalation trial in adults, children, and infants in Kilifi, Kenya. The authors recruited healthy adults and children and used a three-dose primary vaccination series with a booster dose at 1–2 years. Variable doses of R21 and adjuvant (Matrix-M™) were administered at 10µg R21/50 µg Matrix-M™, 5µg R21/25µg Matrix-M™, and 5µg R21/50µg Matrix-M™ to 20 adults, 20 children, and 51 infants. The study is very carefully planned and executed. The immunological methods of analysis are up to date and provide important information.

 i) R21/Matrix-M™ is safe - Self-limiting adverse events were reported relating to the injection site and mild systemic symptoms;

 ii) R21/Matrix-M™ is immunogenic - High levels of IgG antibodies (anti-NANP peptides) were induced when the vaccine was given at varied doses, with the peak immune response seen in infants 28 days after a three-dose primary vaccination series given four weeks apart;

 iii) One of the most relevant pieces of information is that the antibody responses were restored 28 days after a 4th dose given one year post a three-dose primary series in young children and infants.

**Minor comments for authors’ consideration:**

1. **Introduction**: Please add the malaria data provided by the World Malaria Report 2023.
2. **Methods**: The authors should explain why only 0.2 µg/mL was used to coat plates and provide the purity levels of the NANP peptides used.
3. **Results**: The authors should explain if these anti-CSP antibodies are inhibitory in in vitro assays and if they recognize the C-terminal portion of PfCSP.

Is the work clearly and accurately presented and does it cite the current literature?

Yes

If applicable, is the statistical analysis and its interpretation appropriate?

I cannot comment. A qualified statistician is required.

Are all the source data underlying the results available to ensure full reproducibility?

Yes

Is the study design appropriate and is the work technically sound?

Yes

Are the conclusions drawn adequately supported by the results?

Yes

Are sufficient details of methods and analysis provided to allow replication by others?

Partly

Reviewer Expertise:

Malaria vaccine (P. vivax)

I confirm that I have read this submission and believe that I have an appropriate level of expertise to confirm that it is of an acceptable scientific standard.

# Reviewer report: Reviewer response for version 1

**DOI:** 10.21956/wellcomeopenres.21926.r81710

**Published:** 2024-05-24

**Authors:** George Ayodo

**Affiliations:** 1 Jaramogi Oginga Odinga University of Science and Technology, Bondo, Siaya, Kenya

**Abstract**

 a) The background should focus on the R21 not RTS,S/AS01

 b) After change of manufacturing process can be revised as after improved purification process

 The conclusion of the abstract should consider this "evaluated in larger randomised controlled clinical trials to ascertain further safety, reactogenicity, immunogenicity and to assess its efficacy in a broader African paediatric and adult population".

**Introduction**

 Here the focus is on children and adults, and in the abstract, we have adults, children and infants and other part of the paper young children etc.  Can we ensure consistence of the above on the manuscript?

**Methods**

 The authors should include a brief description of the selection of study participants. Was this done in the village or community units etc?

 Why a sample size of 20, 20 and 51 participants?

 Why healthy adults of 18-45 years ( not a much narrow age like <18 or close to 18 years eg 18-24 years) with a sample size of 20? 

 Table 1:- group  females 75% and males 5% this should be corrected

 The age distribution of 18-45 (median of 27 years) of the study participants should be considered as a limitation or may bring some biases

Is the work clearly and accurately presented and does it cite the current literature?

Yes

If applicable, is the statistical analysis and its interpretation appropriate?

Yes

Are all the source data underlying the results available to ensure full reproducibility?

Yes

Is the study design appropriate and is the work technically sound?

Yes

Are the conclusions drawn adequately supported by the results?

Yes

Are sufficient details of methods and analysis provided to allow replication by others?

Partly

Reviewer Expertise:

Epidemiology of malaria

I confirm that I have read this submission and believe that I have an appropriate level of expertise to confirm that it is of an acceptable scientific standard.
